# Supplementary material for: Prophylactic Effect of Bovine Colostrum on Intestinal Microbiota and Behavior in Wild-Type and Zonulin Transgenic Mice
Source: Biomedicines. 2022 Dec 29;11(1):91. doi: 10.3390/biomedicines11010091 (PMC9855927; doi:10.3390/biomedicines11010091)
Supplement: Supplementary file 1 [file biomedicines-11-00091-s001.zip › biomedicines-2087818-supplementary.pdf]

PHYLA	WT *f* BC (%)	WT *f* Ctr (%)	WT *m* BC (%)	WT *m* Ctr (%)	Ztm *f* BC (%)	Ztm *f* Ctr (%)	Ztm *m* BC (%)	Ztm *m* Ctr (%)
p____Firmicutes	56.849	51.591	43.828	49.491	43.568	50.391	43.465	42.935
p____Bacteroidota	40.140	47.742	54.426	49.252	51.512	42.633	49.602	47.986
p____Proteobacteria	0.000	0.010	0.770	0.855	2.378	2.305	4.670	3.499
p____Actinobacteriota	0.092	0.179	0.087	0.134	0.904	1.683	1.284	2.199
p____Campilobacterota	0.000	0.000	0.000	0.000	1.492	1.345	0.747	1.333
p____Verrucomicrobiota	2.918	0.478	0.888	0.268	0.000	0.000	0.073	0.000
p____Desulfobacterota	0.000	0.000	0.000	0.000	0.106	0.693	0.155	1.476
p____Deferribacterota	0.000	0.000	0.000	0.000	0.000	0.931	0.000	0.492
p____Cyanobacteriota	0.000	0.000	0.000	0.000	0.035	0.018	0.000	0.077

FAMILY	WT *f* BC (%)	WT *f* Ctr (%)	WT *m* BC (%)	WT *m* Ctr (%)	Ztm *f* BC (%)	Ztm *f* Ctr (%)	Ztm *m* BC (%)	Ztm *m* Ctr (%)
f____Muribaculaceae	37.478	44.731	54.396	49.251	38.626	34.081	42.265	41.513
f____Lachnospiraceae	29.792	22.400	19.589	13.657	14.813	19.040	10.943	15.319
f____Erysipelotrichaceae	8.075	14.886	7.960	17.619	13.923	14.700	21.633	15.645
f____Lactobacillaceae	0.658	3.220	2.020	1.622	8.326	9.553	4.824	4.029
f____Bacteroidaceae	2.661	3.011	0.000	0.000	5.755	4.044	3.405	3.529
f____Prevotellaceae	0.000	0.000	0.000	0.000	4.522	2.223	2.358	0.314
f____Clostridiaceae	0.523	1.043	0.487	4.892	0.048	0.122	0.058	0.196
f____Oscillospiraceae	7.622	4.332	3.481	2.039	1.518	1.152	1.809	1.732
f____Akkermansiaceae	2.918	0.478	0.888	0.268	0.000	0.000	0.073	0.000
f____Clostridia__UCG-014	2.464	1.186	2.753	3.322	0.067	0.056	0.291	0.144
f____Ruminococcaceae	3.334	1.090	3.275	2.113	0.457	1.626	0.971	0.826
f____Sutterellaceae	0.000	0.01	0.770	0.855	2.378	2.305	4.668	3.499
f____Acholeplasmataceae	1.776	1.232	1.926	1.745	0.951	0.956	1.238	1.936

GENUS	WT *f* BC (%)	WT *f* Ctr (%)	WT *m* BC (%)	WT *m* Ctr (%)	Ztm *f* BC (%)	Ztm *f* Ctr (%)	Ztm *m* BC (%)	Ztm *m* Ctr (%)
g____Turicibacter	8.042	14.826	7.916	17.548	0.168	2.515	0.058	0.163
g____Lachnospiraceae__NK4A136__group	9.344	6.577	4.757	2.657	6.062	7.225	4.138	5.731
g____Faecalibaculum	0.000	0.000	0.000	0.000	2.856	8.509	8.508	12.793

g____Dubosiella	0.000	0.007	0.000	0.000	10.801	3.035	13.045	2.264
g____Parasutterella	0.000	0.010	0.770	0.855	2.378	2.305	4.668	3.499
g____Roseburia	2.115	1.206	0.078	0.000	1.245	5.023	0.102	1.665
g____Anaeroplasma	1.776	1.232	1.926	1.745	0.951	0.956	1.238	1.936
g____Clostridia__UCG-014	2.464	1.186	2.753	3.322	0.067	0.056	0.291	0.144
g____Alloprevotella	0.000	0.000	0.000	0.000	3.843	1.961	2.051	0.000
g____Oscillibacter	3.908	2.205	1.456	0.828	0.786	0.511	0.903	0.518
g____Ruminococcus	1.501	0.693	2.451	1.609	0.274	0.162	0.680	0.023
g____[Eubacterium]__xylanophilum__group	1.701	0.743	1.321	0.715	1.191	0.204	0.831	0.462
g____Helicobacter	0.000	0.000	0.000	0.000	1.492	1.345	0.747	1.333
g____Bifidobacterium	0.000	0.000	0.000	0.000	0.000	1.472	0.662	1.781
g____Desulfovibrio	0.000	0.000	0.000	0.000	0.000	0.491	0.000	1.365
g____Incertae__Sedis	0.794	0.241	0.486	0.223	0.114	1.347	0.226	0.624
g____ A2	0.709	0.222	1.280	0.516	0.125	0.171	0.860	0.000
g____Romboutsia	0.011	0.000	0.249	0.642	0.000	1.236	0.000	0.462
g____Ruminococcaceae	0.017	0.035	1.160	0.116	0.000	0.000	0.000	0.000
g____Blautia	0.140	0.118	0.229	0.148	1.117	0.302	0.269	0.274
g____Lachnospiraceae__UCG-001	0.000	0.000	0.060	0.284	0.000	0.425	0.000	1.095
g____RF39	0.658	0.490	0.620	0.686	0.107	0.145	0.047	0.196
g____Clostridia__vadinBB60__group	0.740	0.591	0.552	0.363	0.100	0.399	0.408	0.282
g____Lachnoclostridium	0.842	0.474	0.424	0.438	0.229	0.128	0.113	0.148
g____Colidextribacter	0.660	0.331	0.450	0.255	0.271	0.215	0.459	0.506
g____Marvinbryantia	0.365	0.591	0.272	0.375	0.765	0.433	0.448	0.604
g____[Eubacterium]__coprostanoligenes__group	0.450	0.438	0.254	0.273	0.000	0.000	0.032	0.000
g____[Eubacterium]__siraeum__group	0.820	0.043	0.070	0.066	0.000	0.000	0.000	0.000
g____Acetatifactor	0.173	0.050	0.169	0.271	0.021	0.061	0.083	0.021
g____GCA-900066575	0.274	0.1333	0.173	0.088	0.098	0.200	0.045	0.260
g____Monoglobus	0.109	0.303	0.137	0.111	0.000	0.000	0.007	0.000

GENUS	WT *f* BC (%)	WT *f* Ctr (%)	WT *m* BC (%)	WT *m* Ctr (%)	Ztm *f* BC (%)	Ztm *f* Ctr (%)	Ztm *m* BC (%)	Ztm *m* Ctr (%)
g____ASF356	0.174	0.118	0.161	0.116	0.167	0.848	0.167	0.358
g____UCG-005	0.210	0.192	0.097	0.100	0.000	0.000	0.000	0.000
g____[Eubacterium]__nodatum__group	0.050	0.075	0.075	0.107	0.059	0.048	0.072	0.101
g____Lactococcus	0.124	0.000	0.120	0.000	0.378	0.062	0.073	0.041
g____Intestinimonas	0.045	0.106	0.107	0.038	0.000	0.000	0.024	0.024
g____Lachnospiraceae__UCG-006	0.000	0.000	0.000	0.000	0.118	0.425	0.000	0.024
g____Tyzzerella	0.000	0.047	0.058	0.133	0.000	0.000	0.000	0.000
g____UCG-010	0.000	0.068	0.037	0.083	0.000	0.000	0.000	0.000
g____Enterorhabdus	0.072	0.144	0.000	0.000	0.121	0.130	0.121	0.346
g____Parvibacter	0.000	0.000	0.052	0.102	0.000	0.000	0.000	0.000
g____Staphylococcus	0.098	0.000	0.000	0.000	0.247	0.046	0.067	0.000
g____Dorea	0.147	0.078	0.000	0.000	0.000	0.000	0.000	0.000
g____Erysipelatoclostridium	0.028	0.012	0.000	0.000	0.592	0.643	0.221	0.509
g____[Eubacterium]__ventriosum__group	0.046	0.051	0.000	0.000	0.000	0.000	0.000	0.000
g____NK4A214__group	0.043	0.024	0.000	0.000	0.000	0.000	0.025	0.017
g____Anaerotruncus	0.061	0.000	0.000	0.000	0.041	0.075	0.020	0.048
g____Erysipelotrichaceae	0.026	0.019	0.000	0.000	0.030	0.000	0.000	0.000
g____Family__XIII__AD3011__group	0.000	0.000	0.000	0.000	0.000	0.041	0.016	0.032
g____UCG-009	0.014	0.020	0.000	0.000	0.030	0.000	0.041	0.059
g____Erysipelatoclostridiaceae	0.000	0.000	0.000	0.000	0.000	0.000	0.011	0.073
g____[Eubacterium]__brachy__group	0.036	0.007	0.000	0.000	0.000	0.000	0.016	0.025
g____Butyricicoccus	0.000	0.000	0.000	0.000	0.070	0.093	0.000	0.112
g____Lachnospiraceae__FCS020__group	0.000	0.000	0.000	0.000	0.000	0.101	0.067	0.117
g____Parabacteroides	0.000	0.000	0.000	0.000	0.994	0.741	0.786	0.664
g____Candidatus__Arthromitus	0.000	0.000	0.000	0.000	0.046	0.119	0.055	0.195
g____Alistipes	0.000	0.000	0.000	0.000	0.648	0.409	0.391	0.832
g____Muribaculum	0.000	0.000	0.000	0.000	0.707	0.313	0.436	0.218

GENUS	WT *f* BC (%)	WT *f* Ctr (%)	WT *m* BC (%)	WT *m* Ctr (%)	Ztm *f* BC (%)	Ztm *f* Ctr (%)	Ztm *m* BC (%)	Ztm *m* Ctr (%)
g____Mucispirillum	0.000	0.000	0.000	0.000	0.000	0.931	0.000	0.492
g____Prevotellaceae__UCG-001	0.000	0.000	0.000	0.000	0.679	0.262	0.307	0.218
g____Odoribacter	0.000	0.000	0.000	0.000	0.290	0.616	0.128	0.774
g____Coriobacteriaceae__UCG-002	0.000	0.000	0.000	0.000	0.632	0.061	0.408	0.144
g____Rikenella	0.000	0.000	0.000	0.000	0.344	0.284	0.080	0.223
g____Rikenellaceae__RC9__gut__group	0.000	0.000	0.000	0.000	0.332	0.229	0.168	0.119
g____Bilofila	0.000	0.000	0.000	0.000	0.084	0.159	0.114	0.062
g____Ureaplasma	0.000	0.000	0.000	0.000	0.112	0.067	0.107	0.162
g____Airococcus	0.000	0.000	0.000	0.000	0.123	0.000	0.000	0.000
g____Peptococcus	0.000	0.000	0.000	0.000	0.000	0.063	0.021	0.103
g____Gastranairophilales	0.000	0.000	0.000	0.000	0.035	0.08	0.000	0.007
g____Rikenellaceae	0.000	0.000	0.000	0.000	0.000	0.000	0.020	0.020
